# Dielectric Huygens’ Metasurface for High-Efficiency Hologram Operating in Transmission Mode

**DOI:** 10.1038/srep30613

**Published:** 2016-07-26

**Authors:** Wenyu Zhao, Huan Jiang, Bingyi Liu, Jie Song, Yongyuan Jiang, Chengchun Tang, Junjie Li

**Affiliations:** 1 Department of Physics, Harbin Institute of Technology, Harbin 150001, China; 2 Key Lab of Micro-Optics and Photonic Technology of Heilongjiang Province, Harbin 150001, China; 3 Beijing National Laboratory for Condensed Matter Physics, Institute of Physics, Chinese Academy of Sciences, P.O. Box 603, Beijing 100190, China

## Abstract

Conventional metasurface holograms relying on metal antennas for phase manipulation suffer from strong Ohmic loss and incomplete polarization conversion. The efficiency is limited to rather small values when operating in transmission mode. Here, we implement a high-efficiency transmissive metasurface hologram by leveraging the recently developed Huygens’ metasurface to construct an electric and magnetic sheet with a transmission efficiency up to 86% and optical efficiency of 23.6%. The high-efficiency originates from the simultaneous excitations of the Mie-type electric and magnetic dipole resonances in the meta-atoms composed of silicon nanodisks. Our hologram shows high fidelity over a wide spectral range and promises to be an outstanding alternative for display applications.

Metasurface holography^1^,^2^,^3^,^4^,^5^ enabling arbitrary wavefront reconstruction and light manipulation has recently attracted considerable research interests and promised bright prospects for optical trapping, quantum optics, and integrated photonics. Taking advantage of the great ability of metasurface to generate abrupt interfacial phase changes beyond the diffraction limit^6^,^7^,^8^,^9^,^10^, it is remarkable among the various computer generated holography systems. Great achievements such as wide-angle projection, elimination of high-order diffraction, and broadband spectral response have been made towards a wide variety of practical applications. For hologram based devices, the performances are closely related to the efficiency of the metasurface, which usually remains a fairly low value for the most existing structures due to the fundamental limits and intrinsic Ohmic loss^11^,^12^. So far, high-efficiency metasurface holograms are usually achieved by combining a top metallic resonator array with a ground metal plane^13^,^14^. This will, however, inevitably increase the structure thickness and limit the operation in reflection mode^4^. Recently, facilitated by the low loss nature, dielectric metasurfaces become promising candidates for high efficiency wave manipulation especially in transmission mode^15^,^16^,^17^,^18^,^19^. The ease to fabrication and fully compatible with standard industrial semiconductor technology could bridge the gap between fundamental nanoscience and practical applications.

In this paper, we demonstrate a high-efficiency transmissive hologram by leveraging the recently developed Huygens’ metasurface. Silicon nanodisks with different radii are chosen as the basic meta-atoms to realize phase modulation due to the low loss nature and simultaneous excitations of electric and magnetic dipole resonances in one element. The 7 level phase map is discretized according to the equidifferent nanodisk radii instead of the equidifferent phase values allowing us to bypass the fabrication difficulty. Experimental results agree very well with the calculations and demonstrate an optical efficiency of 23.6% and transmission efficiency up to 86%. The fidelity of our hologram remains high over a wide frequency range and the full width at the half maximum (FHWM) of the signal to noise ratio (SNR) reaches 55 nm.

## Materials and Methods

**Figure 1:**
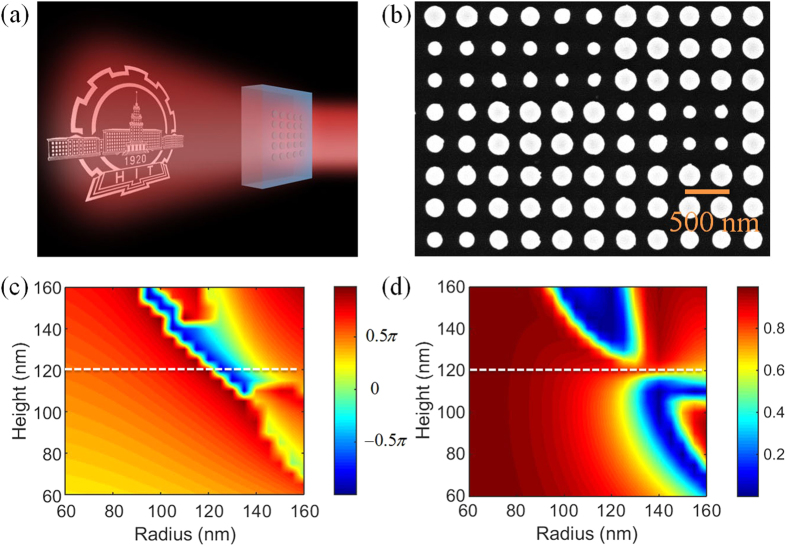
(**a**) The artistic impression and (**b**) SEM image of the Huygens’ metasurface hologram. Silicon nanodisks with different radii are chosen as the building blocks for meta-atoms to realize phase modulation. The nanodisks have the same height of 120 nm and are surrounded by symmetric dielectric environment of glass to enhance transmission efficiency. (**c**) Phase modulation and (**d**) transmission of nanodisk arrays with different heights and radii calculated from numerical simulations. The logo is used with permission from the Harbin Institute of Technology.

Figure 1a,b show the artistic impression and scanning electron microscope (SEM) image of the Huygens’ metasurface hologram, respectively. Silicon nanodisks with different radii are chosen as the basic meta-atom to realize phase modulation in subwavelength scale. Symmetric dielectric environment is introduced to avoid the reflection at the interface of the glass and air, which is also essential to enhance the hologram efficiency. The phase modulation and transmission of the nanodisk arrays with different geometric parameters are calculated using finite-difference-time-domain method software, FDTD solutions from Lumerical Inc. In the simulations, the silicon nanodisks are assumed to be embedded into uniform glass, and the periods of the arrays are fixed at 450 nm both in X and Y directions to ensure polarization-independent. The incident plane wave at 785 nm is normal to the array plane with polarization along the X direction, and periodic condition is used in each boundary of the unit cell to reduce the amount of computation. The permittivity of glass is extracted from the experimental data^20^, and the complex refractive index of silicon is measured by ellipsometer (Figure S3 in supporting information).

**Figure 2:**
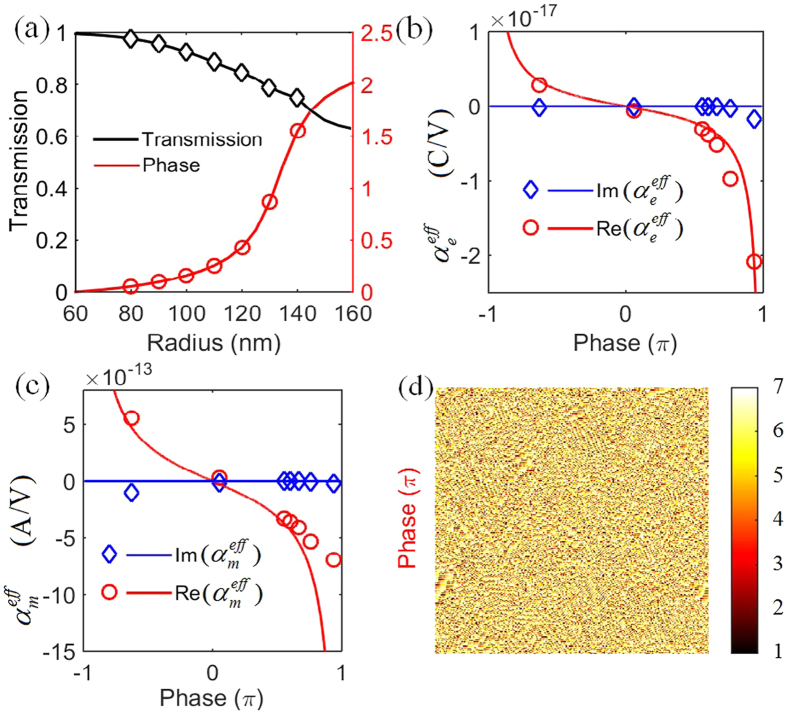
(**a**) Phase modulation and transmission for different nanodisk radii while the nanodisk height is fixed at 120 nm. The phase modulation of 60 nm radius is set to be zero and all negative phases are changed to positive by adding 2*π*. The phase modulation and transmission of the chosen radii for the basic discrete elements of the hologram are indicated by circles and diamonds, respectively. The radii of the 7 level phase elements vary from 80 nm to 140 nm with an increment of 10 nm. (**b**) Surface electric polarizability and (**c**) magnetic polarizability for different phase modulation. The solid lines are calculated for an ideal Huygens’ metasurface while the circles and diamonds illustrate the actual values of the silicon nanodisks. (**d**) Phase map of the hologram retrieved from an adaptive GS algorithm.

Figure 1c,d show the phase modulation and transmission of the nanodisk arrays with different heights (H) and radii (R). For the nanodisk height of 120 nm, full phase coverage (0−2*π*) and relatively high transmission can be achieved at the same time by varying the nanodisk radius. Figure 2a plots the phase modulation and transmission for different nanodisk radii while the height is fixed at 120 nm. Here for convenience, the phase modulation of 60 nm radius is set to be zero and all negative phases are changed to positive by adding 2*π* to them.

The underlying physical mechanism of the phase modulation is related to the excitation of optical Huygens’ sources^21^,^22^. Huygens’ principle, treating each point on a wavefront as a secondary source of outgoing waves, is developed by Love and Schelkunoff to specify the secondary sources in terms of fictitious electric and magnetic currents fulfilling the rigorous boundary condition^21^. By exquisitely constructing the electric and magnetic currents on the surface, arbitrary prescribed distribution of field on either side can be engineered at will. In Huygens’ metasurface, certain equivalent electric and magnetic currents are obtained by designing electric and magnetic dipole resonances with suitable surface polarizabilities (Figure S1 in supporting information). Figure 2b,c show the required surface electric and magnetic polarizabilities for an ideal Huygens’ metasurface with full phase control and 100% transmission calculated from theoretical expressions^23^,


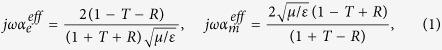


where *ω* is the angular frequency, *μ* and *ε* are the permeability and permittivity of the free space, and *R* and *T* are the complex reflection and transmission coefficients, respectively. Silicon nanodisk is a promising candidate because of its simultaneous excitations of both electric and magnetic dipole resonances in only one element^24^,^25^,^26^,^27^. Also, unlike plasmonic metasurfaces, silicon structures facilitate high transmission, low losses, and compatibility with existing semiconductor technologies^28^,^29^. The surface polarizabilities of silicon nanodisks are also indicated by circles and diamonds in Fig. 2b,c. As the radius is the only freedom of the geometric parameters to tailor the electric and magnetic resonances, the actual polarizabilities of the meta-atoms are slightly deviated from the ideal values, which mainly causes a decrease in transmission as illustrated by the black line in Fig. 2a. The chosen radii for the basic discrete elements of the hologram cover from 80 nm to 140 nm with an increment of 10 nm as shown in Table 1. This is different from the previous work where the phase is discretized according to the equidifferent phase values. In [26] the strong dependence of the phase modulation on the geometric size requires the smallest radius step of nanodisks near 3 nm, which strongly challenges today’s fabrication technology, thus, precluding the direct experimental verification of such optical Huygens’ surface. Here, the phase is discretized according to the equidifferent nanodisk radii instead of the equidifferent phase values allowing us to bypass the fabrication difficulty and implement a 7 level phase map.

The phase map of the hologram is extracted using an adaptive Gerchberg–Saxton algorithm^30^ (Figure S2 in supporting information). (i) The iteration starts in the image plane with a random phase and an amplitude of the goal image. (ii) The wavefront is then propagated back to the hologram plane using inverse Fresnel diffraction. In the hologram plane, the phase map is extracted and discretized according to the phase modulations of the 7 elements from the look-up table. (iii) The new wavefront in the hologram plane calculated with the phases getting from Table 1 and unit amplitude is propagated to the image plane through Fresnel diffraction. (iv) The phase of the new wavefront in the image plane is kept unchanged while the amplitude is forced to be the amplitude of the goal image. One iteration contains the successive processes from (2–4), and 20 iterations are sufficient to get a convergent phase map. Figure 2d shows the calculated phase map after 50 iterations, the goal image is the badge of Harbin Institute of Technology (HIT), and the image plane is set to be 150 mm away from the hologram plane.

As demonstrated in the previous text, the phase modulation and transmission of each nanodisk meta-atom are calculated by performing simulation of a cell with periodic boundary condition, which assumes the structure comprises infinite periodic nanodisk array. As the response of the nanodisk is strongly affected by their coupling to adjacent neighbors, the phase response of a meta-atom is accurate only when its neighbors have the same radius. However, in the hologram phase map shown in Fig. 2d, the 7 level elements are irregularly distributed with most pixels surrounding by different neighbors. The effects between the coupling of nanodisks with their different neighbors will change the phase modulation and transmission making them deviate from the optimal design, which is detrimental for the image quality. The correction of the asymmetric coupling effects through optimizing the whole phase map with numerical calculations is impossible for the limited computation resources. However, the coupling effects and fabrication imperfections can be well interpreted by introducing a random phase noise on the phase map seen in Figure S5 in supporting information. In order to decrease the coupling effects, here, a 2 × 2 periodic arrangement is adopted, where a pixel of hologram is represented by 4 nanodisks. For such arrangement, every nanodisk is surrounded by at least 2 same neighbors, and a weakened asymmetric coupling effect can be envisioned compared with the 1 × 1 arrangement.

To demonstrate the hologram experimentally, a layer of polycrystalline silicon film (120 nm) is deposited using plasma enhanced chemical vapor deposition method (PECVD) on a glass substrate. The hologram with footprint of 450 μm × 450 μm are then fabricated using standard electron beam lithography followed by ion beam etching process. The samples are, then, sandwiched between two glass slides with a 31μm spacer, and the matching liquid with refractive index of 1.45 is infiltrated under vacuum environment through capillary action in order to remove the air bubbles. In order to capture the reconstructed image, the collimated 785 nm light from a diode pumped solid-state laser is normally incident on the hologram. The diameter of the laser beam is around 1.5 mm, which fully covers the hologram. The transmitted holographic image is projected onto a white screen 150 mm away from the sample, which is then captured with a gray charge coupled device (CCD) camera. In order to measure the transmission efficiency defined by the transmitted power divided by the incident power, the incident beam is focused on a spot size slightly smaller than the fabrication area by an objective (5X). For the 785 nm laser incident, the transmitted light is collected by a power meter. As for the halogen lamp source, on the other side the light is collected by another 20X objective and transmitted to an optical fiber spectrometer (USB2000+, Ocean Optics). The optical efficiency, which is defined as the ratio between the optical power projected into the image region and the input power, is also determined using the same method in [4].

## Results and Discussion

**Figure 3:**
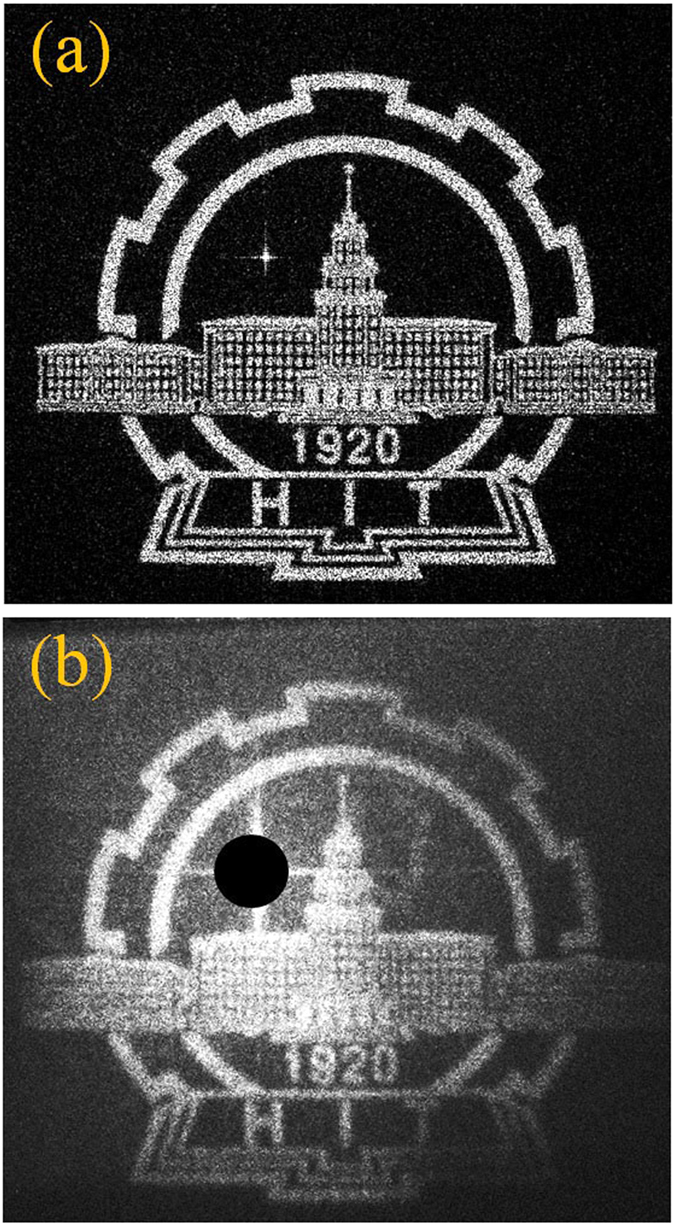
(**a**) Calculated hologram based on a 2 × 2 periodic arrangement. The school badge of HIT with resolution of 500 × 500 pixels is set to be the goal image and the distance between the hologram plane and image plane is 150 mm. The 500 × 500 pixels’ phase map is fabricated on a 450 × 450 *μ*m^2^ footprint. (**b**) Holographic image captured in experiment and the zero order diffraction (bright spot) is blocked to avoid disturbance to the image. In order to avoid pattern distortion, the goal image is pre-compensated with a wide angle correction.

One of the distinct advantages of such metasurface hologram is the wide angular range allowing large projection area and view angle. The angular range of the reconstructed image in the far field, *α*, can be calculated according to Δ*P* = *λ*/(2 tan(*α*/2)). Where *λ* is the operating wavelength, and Δ*P* is the pixel size of the hologram. The angular range of the hologram is 47°. Because of the large angular range, a more rigorous Rayleigh–Sommerfeld diffraction method is used to reconstruct the holographic image, and the goal image is pre-compensated with a wide angle correction. Figure 3a shows the reconstructed image from calculation, and the badge of HIT is clearly discernable. Figure 3b shows the captured image in experiment which is in excellent agreement with the calculation. The slight distortion originates from the deviation of the phase modulations from optimal values due to the asymmetric coupling effects between the hologram pixels and the fabrication imperfection.

**Figure 4:**
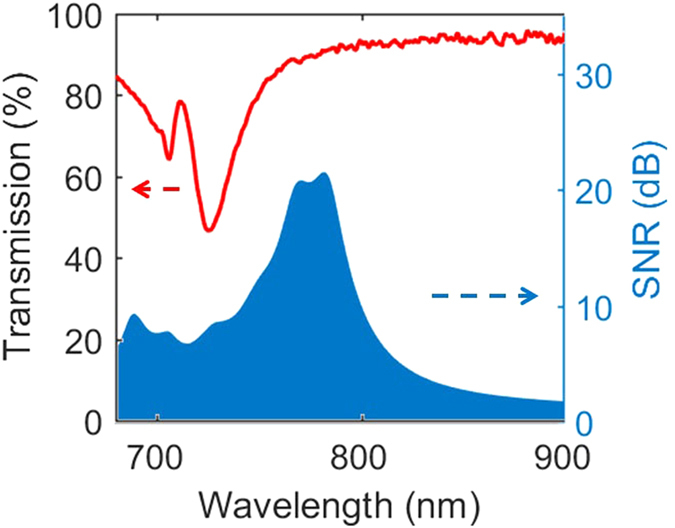
Transmission and SNR of the holographic image as a function of incident wavelengths.

**Figure 5:**
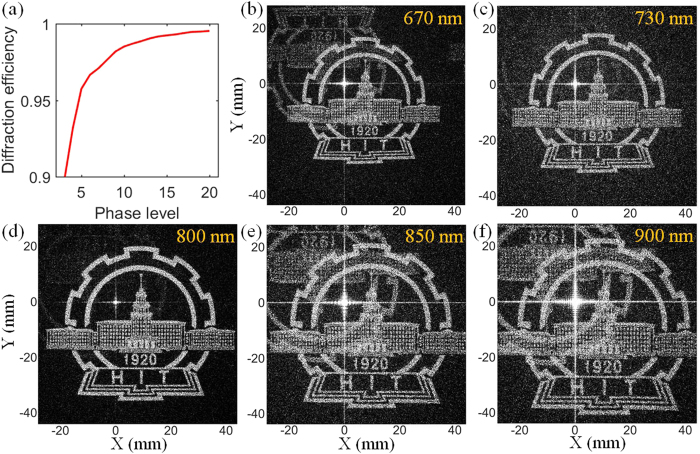
(**a**) Diffraction efficiency of the goal image as a function of different phase levels. (**b–f**) Holographic images reconstructed under different incident wavelengths. The images are normalized with respect to the maximum intensity of image at 785 nm.

The measured transmission efficiency for 785 nm laser is 86%, and the transmission spectrum of the hologram is shown in Fig. 4. The metasurface hologram remains a high transmission efficiency over a wide frequency range. The optical efficiency for 785 nm laser is 23.6% indicating most transmitted energy is funneled to the zero-order speck. The main reasons are two folds: first, the amplitude manipulations for different meta-atoms are not identical (Table 1) due to the radius is the only degree of freedom to tailor the resonances of the nanodisks. Second, the asymmetric coupling between different meta-atoms will make both the phase and amplitude manipulations deviate from the optimal design. Actually, we have tried the 1 × 1 periodic arrangement, where a pixel of hologram is represented by one nanodisk and strong asymmetric coupling effects will happen between different meta-atoms. The measured holographic image is nothing but background noise and one cannot even observe the outline of the goal image (Figure S7 in supporting information). The phase discreteness will also, to some extent, decrease the optical efficiency. However, the influence is limited for our 7 phase level design. Figure 5a depicts the diffraction efficiency of the goal image as a function of different phase levels calculated from the diffraction theory^31^. The diffraction efficiency grows dramatically as the phase level increases at the very beginning and then varies slightly for phase level exceeding 6.

The spectral response of the hologram under different operating wavelengths is also studied using the Rayleigh–Sommerfeld diffraction method. Figure 5b–f show the different reconstructed images at different incident wavelengths. The hologram can generate goal image over a broad spectral range, and as the hologram operates away from the optimal wavelength (785 nm), the image quality is gradually degraded by the background noise. For the wavelength deviation exceeding 55 nm, the twin image becomes evident and superimposes on the goal image due to the unintended amplitude and phase modulation. Such dispersion effects are limited by the resonant nature of the meta-atoms. Removing the twin image and multiple diffraction orders require a reasonable design of wide band metasurface with subwavelength pixel size^5^,^17^, which cannot be fulfilled by our present design. However, a more straightforward way to reduce the twin image influence is to arrange the target image in a corner of the whole hologram. The twin image is centrosymmetric to the target holographic image. So, even though the twin image still exists, it will not superimpose on the target holographic image and decrease the fidelity. One can also notice that the size of image increases as the wavelength increases due to the growing angular range. The hologram is inherently dispersive due to the resonant nature of the electric and magnetic dipole resonances of the meta-atom and the strong dependence of angular range on operating wavelength. Figure 4 gives the efficiencies and the SNA evolution with the change of operating wavelengths. The SNA is calculated by^17^,


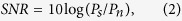


where *P*_*s*_ is the peak intensity in the reconstructed image, *P*_*n*_ is the standard deviation of the background noise. The SNR reaches its highest value of 21.5 at 785 nm, and as the working wavelength deviates from 785 nm, the phase modulation shift from the optimal design resulting in a degraded image quality with a FWHM of 55 nm.

## Conclusion

In summary, we take advantage of the high efficiency of Huygens’ surface to realize optical metasurface hologram with a transmission efficiency up to 86% and optical efficiency of 23.6% capable of operating in transmission mode. Silicon nanodisk supporting the simultaneous excitations of electric and magnetic dipole resonances in only one element is adopted as the basic meta-atom of the hologram to realize phase manipulation. Measured holographic image from experiments are in excellent agreement with the calculations using the rigorous Rayleigh–Sommerfeld diffraction method. The spectral response analysis shows our hologram keeps a high fidelity over a wide frequency range and the FWHM of the SNR reaches 55 nm. Such hologram possessing the merits of high efficiency, wide angular range, polarization-independent, and easy fabrication is ideal for future display applications.

## Additional Information

**How to cite this article**: Zhao, W. *et al*. Dielectric Huygens’ Metasurface for High-Efficiency Hologram Operating in Transmission Mode. *Sci. Rep.* **6**, 30613; doi: 10.1038/srep30613 (2016).

## Supplementary Material

Supplementary Information

## Figures and Tables

**Table 1:** Look-up table of phase modulation and transmission of the 7 level phase elements.

Radius (nm)	80	90	100	110	120	130	140
Phase (π)	0.05	0.10	0.16	0.25	0.43	0.87	1.55
Transmission	0.98	0.96	0.93	0.89	0.85	0.79	0.75
