# Supplementary material for: Dielectric Huygens’ Metasurface for High-Efficiency Hologram Operating in Transmission Mode
Source: Sci Rep. 2016 Jul 26;6:30613. doi: 10.1038/srep30613 (PMC4960531; doi:10.1038/srep30613)
Supplement: Supplementary Information [file srep30613-s1.pdf]

Supplementary Material for Dielectric Huygens' Metasurface for High-Efficiency Hologram Operating in Transmission Mode

Wenyu Zhao,[1+] Huan Jiang,[1+] Bingyi Liu,[1] Jie Song,[1,2] Yongyuan Jiang,[1,2,*] Chengchun Tang,[3] and Junjie Li[3]

[1]*Department of Physics, Harbin Institute of Technology, Harbin 150001, China*

[2]*Key Lab of Micro-Optics and Photonic Technology of Heilongjiang Province, Harbin 150001, China*

[3]*Beijing National Laboratory for Condensed Matter Physics, Institute of Physics, Chinese Academy of Sciences, P.O. Box 603, Beijing 100190, China*

[*]Corresponding author: jiangyy@hit.edu.cn

[+]These authors contributed equally to this work.

1. Phase modulation of Huygens' Metasurface

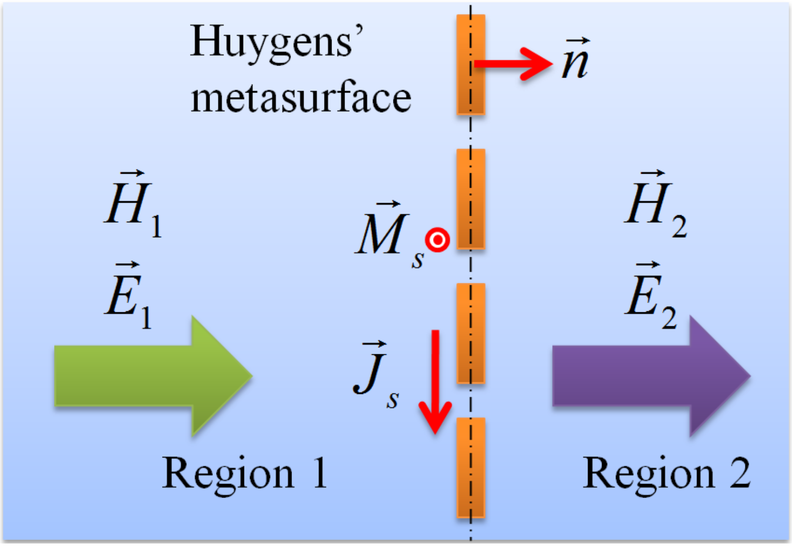

Figure S1. Schematic of the Huygens' metasurface. The metasurface satisfies the boundary condition between region 1 and 2 by constructing equivalent electric and magnetic surface currents.

Figure S1 shows the schematic of the Huygens' metasurface, a plane wave is normally incident on the metasurface and transmitted with arbitrary wavefront. According to the Huygens' principle developed by Love and Schelkunoff, fictitious electric and magnetic surface currents can be introduced to fulfill the boundary conditions,

$$\vec{J}_s = \vec{n} \times (\vec{H}_2 - \vec{H}_1), \quad \vec{M}_s = -\vec{n} \times (\vec{E}_2 - \vec{E}_1). \tag{1}$$

The surface currents can be related to the surface electric and magnetic polarizabilities,

the polarizabilities are defined as the ratio of the dipole moment to the local field,

$$\vec{J}_s = j\omega\alpha_e^{eff} \cdot \vec{E}_{av}, \quad \vec{M}_s = j\omega\alpha_m^{eff} \cdot \vec{H}_{av}. \tag{2}$$

where α_e^{eff} and α_m^{eff} are the electric and magnetic polarizabilities, $\vec{E}_{av}$ and $\vec{H}_{av}$ represent the average electric and magnetic fields tangential to the surface, ω is the angular frequency. The complex reflection (R) and transmission coefficients (T) can then be calculated in terms of polarizabilities,

$$j\omega\alpha_e^{eff} = \frac{2(1-T-R)}{(1+T+R)\sqrt{\mu/\varepsilon}}, \quad j\omega\alpha_m^{eff} = \frac{2\sqrt{\mu/\varepsilon}(1-T+R)}{(1+T-R)}. \tag{3}$$

where μ and ε are the permeability and permittivity of the free space. As demonstrated in equation (3), arbitrary phase modulation and unit transmission coefficients can be achieved by constructing the electric and magnetic sheet with suitable polarizabilities. Figure 2b and c in the main text show the electric and magnetic polarizabilities for an ideal Huygens' metasurface.

2. Wave propagation using Fresnel diffraction method

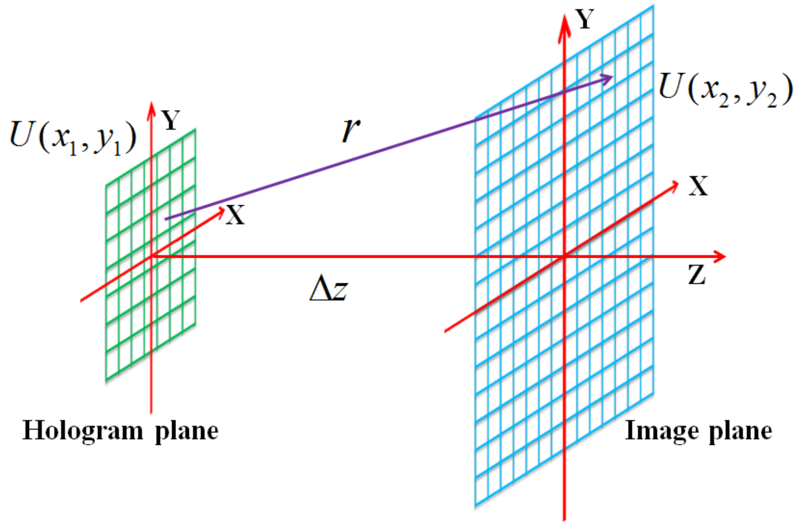

Figure S2. Diagram of the wave propagation from hologram plane to image plane.

The adaptive Gerchberg–Saxton (GS) algorithm is based on Fresnel diffraction and inverse Fresnel diffraction. The Fresnel diffraction is conducted in the form of Fourier transform in order to speed up the computation process. Figure S2 shows the diagram of the wave propagation from hologram plane to image plane. The Fresnel diffraction integral can be mathematically expressed as,

$$U(x_2, y_2) = \frac{e^{jk\Delta z}}{j\lambda\Delta z} e^{j\frac{k}{2\Delta z}(x_2^2+y_2^2)} \int_{-\infty}^{\infty}\int_{-\infty}^{\infty} U(x_1, y_1) e^{j\frac{k}{2\Delta z}(x_1^2+y_1^2)} e^{j\frac{2\pi}{\lambda\Delta z}(x_1x_2+y_1y_2)} dx_1 dy_1 . \tag{4}$$

Equation (4) can be rewritten in the form of Fourier transform by multiplying the source field by a quadratic phase.

$$U(x_2,y_2)=\frac{e^{jk\Delta z}}{j\lambda\Delta z}e^{j\frac{k}{2\Delta z}(x_2^2+y_2^2)}\mathrm{FFT}\{U(x_1,y_1)e^{j\frac{k}{2\Delta z}(x_1^2+y_1^2)}\}, \quad (5)$$

which then can be calculated by Matlab using built-in fast Fourier transform function. In the same way, the inverse Fresnel diffraction can also be rewritten in the form of inverse Fourier transform and calculated by inverse fast Fourier transform function,

$$U(x_1,y_1)=\mathrm{IFFT}\{U(x_2,y_2)\,j\lambda\Delta z e^{-jk\Delta z}e^{-j\frac{k}{2\Delta z}(x_2^2+y_2^2)}\}e^{-j\frac{k}{2\Delta z}(x_1^2+y_1^2)}. \quad (6)$$

3. Wave propagation using Rayleigh–Sommerfeld diffraction method

As demonstrated in the previous text, due to the large angular range, Fresnel diffraction method is not accurate enough to reconstruct the holographic image, and a more accurate Rayleigh–Sommerfeld diffraction method is adopted. The Rayleigh–Sommerfeld integral can be expressed as,

$$\begin{aligned}&U(x_2,y_2)=\iint U(x_1,y_1)\frac{e^{jkr}}{2\pi r}\frac{\Delta z}{r}(\frac{1}{r}-jk)dx_1dy_1\\&r=\sqrt{(x_2-x_1)^2+(y_2-y_1)^2+\Delta z^2}\end{aligned} \quad (7)$$

It is the rigorous integral formula without any approximation, which can be used for both near and far field region. The diffraction integral is calculated by direct numerical integration. On the hologram plane, the wavefront is sampled to N×N equidistant grids, where N is the hologram resolution. For a point on the image plane, the $U(x_{2m},y_{2m})$ can be calculated by numerical integration as a Riemann sum,

$$\begin{aligned}&U(x_{2m},y_{2m})=\sum_{i=1}^{N}\sum_{n=1}^{N}U(x_{1i},y_{1n})\frac{e^{jkr}}{2\pi r}\frac{\Delta z}{r}(\frac{1}{r}-jk)\Delta x\Delta y.\\&r=\sqrt{(x_{2m}-x_{1i})^2+(y_{2m}-y_{1n})^2+\Delta z^2}\end{aligned} \quad (8)$$

4. Methods for spectral response analysis

In order to analyze the spectral response of the hologram, the phase modulation and transmission of the nanodisks under different wavelengths are calculated using FDTD Solutions. The complex refractive index of polycrystalline silicon is measured using an ellipsometer, GES5E from Semilab and fitted automatically by the build-in Cauchy model. The imaginary part of the refractive index is relatively low in the interested frequency range (Figure S3). Figure S4 a and b show the spectral response of the phase modulation and transmission under different nanodisk radius. As the operating wavelength deviates from optimal design (785 nm), both the phase modulation and transmission vary dramatically. The extracted phase modulation and transmission are combined to form the new wavefront on the hologram plane which is then calculated to reconstruct the holographic image using Rayleigh–Sommerfeld diffraction method.

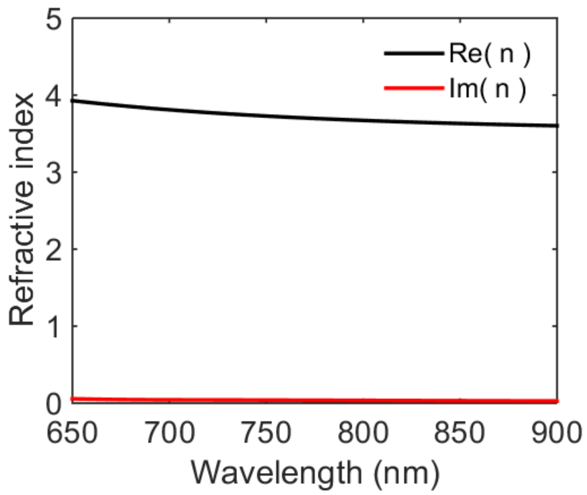

Figure S3. The complex refractive index of polycrystalline silicon film.

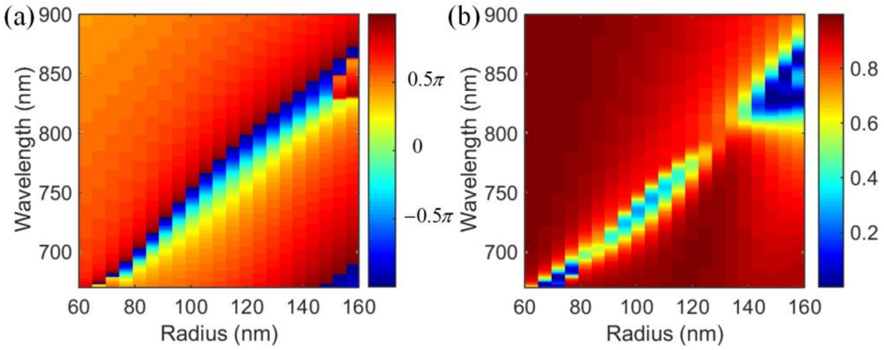

Figure S4. Spectral response of phase modulation and transmission.

5. Modeling of fabrication imperfection

The phase modulation and transmission are extracted using FDTD Solutions. In the simulations, periodic boundary conditions are applied to the unit cell to reduce the computation resources, which assumes the structure comprises infinite nanodisk array. For the hologram map, most nanodisks are located in asymmetric neighbor environment and the actual phase modulation and transmission will deviate from the optimal design. The optimizing of the whole phase map using FDTD simulations to correct the asymmetric coupling effects is impossible for the available computation resources. However, the coupling effects and fabrication imperfections can be well understood by adding a random phase noise on the phase map. Figure S5 shows the holographic image with different phase noise. The goal image can be well reproduced by the 7 level phase map without phase noise. As the phase noise increases, the holographic image gradually loses details. It is quite surprising that the hologram is very robust against the phase noise. Even for a phase noise up to 0.8π, the goal image is clearly discernable. In the case of phase noise up to 2π, all the useful information of the phase map is immersed into noise and the hologram is more like a ground glass. Figure S6 also gives the SNR evolution under different phase noise level.

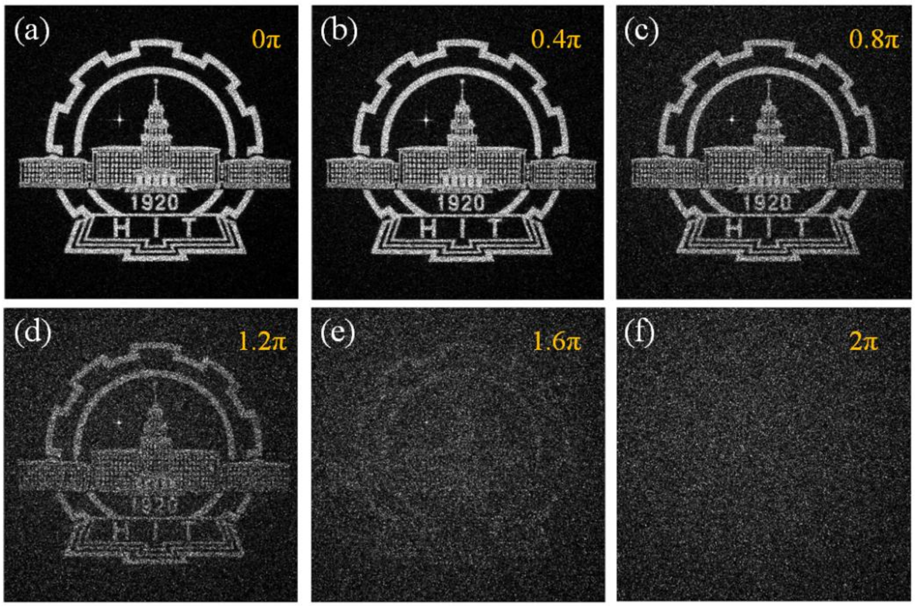

Figure S5. Holographic image reconstructed from the phase map with different noise level. The logo is used with permission from the Harbin Institute of Technology.

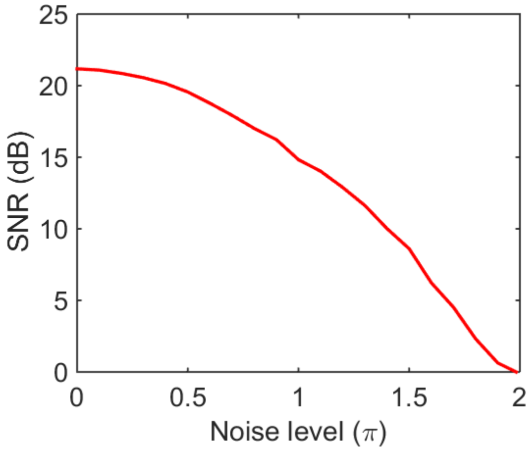

Figure S6. The SNR of the holographic images under different noise level.

6. Analysis of the twin image issue

The twin image is originated from the unintended phase and amplitude modulations. The employed meta-atom relies on the resonant nature for wave manipulation which is inherently dispersive. As the operating wavelength departs from 785 nm, both the phase and amplitude modulations will deviate from the optimal design and result in a centrosymmetric twin image. The fabrication imperfection and asymmetric coupling effects will also generate unintended phase and amplitude manipulations and this is more obvious in a 1×1 arrangement. Figure S7 shows the scanning electron microscope (SEM) image of metasurface hologram with 1×1 arrangement where a pixel of hologram is represented by one nanodisk. The measured holographic image is nothing but background noise and one cannot even observe the outline of the target image.

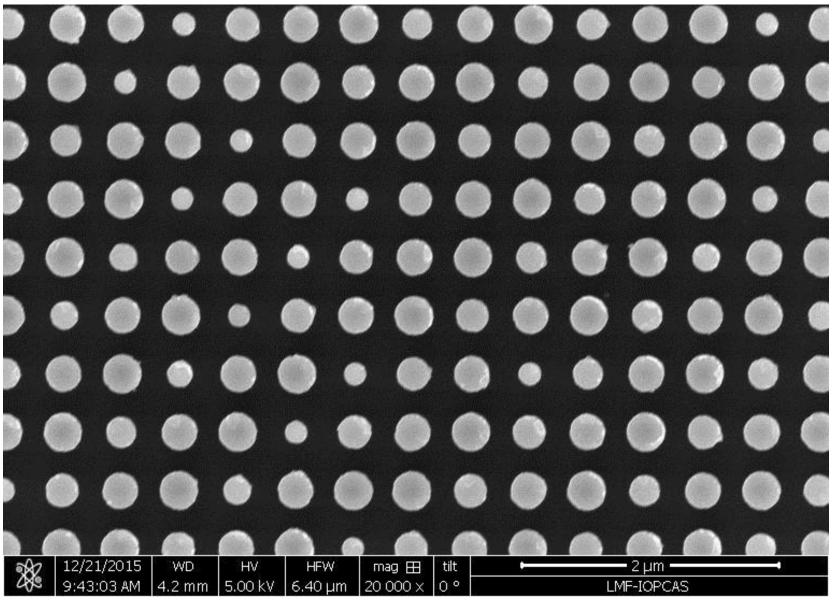

Figure S7. SEM image of the metasurface hologram with 1×1 arrangement.
